# O01 Auditing the duration of antibiotic therapy for children treated as inpatients for community acquired pneumonia

**DOI:** 10.1093/jacamr/dlac133.001

**Published:** 2023-01-19

**Authors:** Emma Alexander, Dharini Chandrasegaran, Deborah Burns, Cauvery Pal

**Affiliations:** Children’s Services, Homerton University Hospital, Homerton Row, London, E9 6SR, UK; Children’s Services, Homerton University Hospital, Homerton Row, London, E9 6SR, UK; Children’s Services, Homerton University Hospital, Homerton Row, London, E9 6SR, UK; Children’s Services, Homerton University Hospital, Homerton Row, London, E9 6SR, UK

## Abstract

**Background:**

Antibiotics are commonly prescribed for children with respiratory symptoms when a bacterial infection is suspected, or the patient is felt to be at higher risk of complications. However, in the context of the significant threat posed by antimicrobial resistance, it is vital for clinicians to avoid over-prescription. In November 2021, the CAP-IT randomized clinical trial reported that lower-dose and shorter duration prescriptions (3 days) of amoxicillin for community acquired pneumonia (CAP) was noninferior to higher-dose and longer-duration (7 days), with regards to the primary outcome of later need for antibiotic re-treatment. Consequently, we wanted to audit the prescribing habits for inpatient children at our hospital in relation to our trust guidelines, which at the time recommended 5–7 days of amoxicillin in simple cases of CAP.

**Methods:**

We conducted a retrospective audit of children treated with antibiotics for a community acquired pneumonia on the inpatient ward of our district general hospital, between 1 November and 15 December 2021. Audit registration number was 3204–3892. We collected information on antibiotics prescribed, route of administration, allergies, duration of antibiotics, infection markers, febrile episodes, oxygen requirement, past medical history and details of X-ray performed.

**Results:**

We identified a total of 30 children within the time period fulfilling inclusion criteria with a median age of 2 years (range 2 months–12 years). 12 (40%) of these children received at least one dose of IV or IM antibiotics, 18 (60%) received oral antibiotics only. 12 (40%) received amoxicillin only, 8 (26.7%) received co-amoxiclav only, and the remainder (33.3%) received a combination of antibiotics. No child had any allergy to antibiotics. The median duration of the course of antibiotics (including any prescribed as a TTA) was 7 days (range 1–9), and seven children (23.3%) received greater than 7 days of antibiotics. The median length of stay in hospital was 2 days (range 1–8 days). The median peak white cell count was 12.05, median CRP was 44. Overall 20 children (66%) required oxygen, and 6 children (20%) required high-flow oxygen. Eleven (36.7%) children were never febrile during their admission and only 3 (10%) of children had a severe underlying chronic disease. 28 (93.3%) of children had a chest X-ray performed, of which nine (32.1%) had no focal changes on the formal X-ray report, despite all receiving antibiotics.

**Conclusions:**

From this audit, we identified that many children are receiving prolonged courses of antibiotics for community acquired pneumonia (7 days or more) and many are receiving antibiotics despite no focal changes identified on chest X-ray. Prescribing decisions are complex and relate to many factors including the degree of clinical concern. However, we believe this audit identified a degree of over-prescribing, particularly in the context of the results of the CAP-IT trial. Following this audit we implemented a change in trust guidelines via Paediatric Microguide, allowing prescription of a 3 day course of amoxicillin for appropriate cases of CAP. It is our aim that this will reduce over-prescribing and hence improve antimicrobial stewardship in future, with a re-audit planned for Winter 2022–23.

